# RETRACTION: Sonographic Comparison of Subcutaneous Fat Layer Thickness in the Scalp Area in Patients With Androgenetic Alopecia Compared to Healthy Individuals: Cross‐Sectional

**DOI:** 10.1111/srt.70224

**Published:** 2025-09-14

Tabatabaiei MR, Ghassemi M, Mohammadi Z, Toufani S, Farshad K, Sadeghzadeh Bazargan A. Sonographic comparison of subcutaneous fat layer thickness in the scalp area in patients with androgenetic alopecia compared to healthy individuals: Cross‐sectional. *Skin Res Technol*. 2024;30(7):e13837. doi:10.1111/srt.13837

The above article, published online on 4 July 2024 in Wiley Online Library (wileyonlinelibrary.com), has been retracted by agreement between *Skin Research and Technology*; and John Wiley & Sons Ltd. The article was accepted for publication following an insufficient peer review process that does not meet the standards of the journal's peer review policy. Furthermore, Figures 1 and 2 have been sourced from previously published articles from different authors without adequate permissions and citations. Finally, the article lacks important information to interpret and reproduce the findings. Accordingly, the article has been retracted. The authors have been informed of this decision but were unavailable for final confirmation.

